# Exploring the Driving Factors of Construction Industrialization Development in China

**DOI:** 10.3390/ijerph15030442

**Published:** 2018-03-03

**Authors:** Xiaer Xiahou, Jingfeng Yuan, Yan Liu, Yuchun Tang, Qiming Li

**Affiliations:** 1 Department of Construction Management and Real Estate, School of Civil Engineering, Southeast University, Nanjing 211189, China; xh@seu.edu.cn (X.X.); 101011337@seu.edu.cn (J.Y.); tangyuchunseu@163.com (Y.T.); 2 Faculty of Civil Engineering and Geosciences, Delft University of Technology, Stevinweg 1, 2628 CN Delft, The Netherlands; y.liu-9@tudelft.nl

**Keywords:** construction industrialization (CI), driving factors, grounded theory method, content analysis, pull and push, questionnaire survey, China

## Abstract

Construction industrialization (CI) has been adopted worldwide because of its potential benefits. However, current research shows the incentives for adopting CI may differ in different regions. While the promotion of CI in China is still at the initial stage, a systematical analysis of the driving factors would help decision makers get a comprehensive understanding of CI development and select proper strategies to promote CI. This research combines qualitative and quantitative methods to explore the construction industrialization driving factors (CIDFs) in China. The grounded theory method (GTM) was employed to explore CI concepts among 182 CI-related articles published in 10 top-tier journals from 2000 to 2017. A total of 15 CIDFs were identified, including one suggested by professionals during a pre-test questionnaire survey. The analysis showed that the development of CI in China is pushed by macrodevelopment and pulled by the government and is also a self-driven process. The major driving factors for CI adoption in China are the transformation and upgrade of the conventional construction industry and the solution of development dilemmas. Our study also suggests that pilot programs are, currently, the most effective method to promote CI in China and to accumulate experience so to gain recognition by the society. This research is also of value for CI promotion in other developing countries.

## 1. Introduction

Construction industrialization (CI) together with its various synonymous terms, such as industrial building (IB), prefabrication, pre-assembly, modern method of construction, off-site manufacturing, off-site production, and off-site construction (OSC), has been widely adopted as an alternative to conventional site-based construction around the world for a long period [1,2,3]. By manufacturing the construction components in the factory and then assembling them in the construction site, the adoption of CI would help to save cost and time, improve quality, reduce the consumption of resource and the production of waste, increase labor efficiency to meet labor shortage, and offer a healthier and safer work environment for construction workers [3,4,5,6,7]. The development of CI would also enhance the performance of construction projects of various types [4,8], therefore it is considered to be a critical approach for the upgrade of the conventional construction industry, especially for developing countries like China [1,3,6,9,10]. The implementation of CI has been listed as the top-level design for the development and modernization of construction industry by the Chinese government [1,3,9,10].

To promote the implementation of CI, barriers and risks have been studied by scholars [3,6,11,12]. Blismas et al. [13] revealed that skills shortages and lack of adequate OSC knowledge are generally the greatest issues facing off-site construction in Australia. Yunus and Yang [14] identified 18 sustainability factors, including time of construction, production, etc., which are critical for IB system implementation in Malaysia. Research conducted in China by Mao et al. [1], Zhang et al. [3], Zhang et al. [9], and Tam et al. [15] also echoed these results. Luo et al. [11] also studied the risk factors affecting practitioners’ attitudes towards the adoption of CI in China. In the meantime, there also exist opportunities for the development of CI. With the progresses of science and technologies, a number of management methods and advanced technologies have been introduced into the construction industry to help the adoption of CI [16,17,18,19,20,21]. Recently, rapid urbanization campaigns in developing countries like China have boosted the demands for more dwellings [7,8,22]. Social and environmental constraints, such as the labor shortage, resource scarcity, and the concerns for the lifecycle performance of construction products, also urge the decision makers to adopt the construction methods of CI [23,24]. However, the adoption of CI in China is much slower than expected [3,10,15]. To find solutions to these problems and accelerate CI implementation, it is essential to understand the driving forces of CI development in China. A systematical understanding of driving factors of CI development in China would improve the cognition of all aspects and provide valuable references for the decision makers to select proper strategies to promote CI in China.

The goal of this paper is to identify the critical driving factors of CI development in China. The findings would help all stakeholders to improve their understanding of CI. In addition, the identified driving factors could also be used as significant references for decision makers to select proper strategies to ensure the efficiency and effectiveness of CI development in China. The findings are also beneficial to developing countries with a similar background.

The remainder of this paper is organized as follows. Section 2 illustrates the research methods of this study. Content analysis targeted at exploring construction industrialization driving factors (CIDFs) within the construction industry is conducted in Section 3 with the help of the grounded theory method (GTM). Section 4 conducts a structured questionnaire survey in Jiangsu province, China, to investigate the views on the identified driving factors. The results of this research are presented and discussed in Section 5. Finally, the conclusions are stated.

## 2. Research Methods

**Figure 1:**
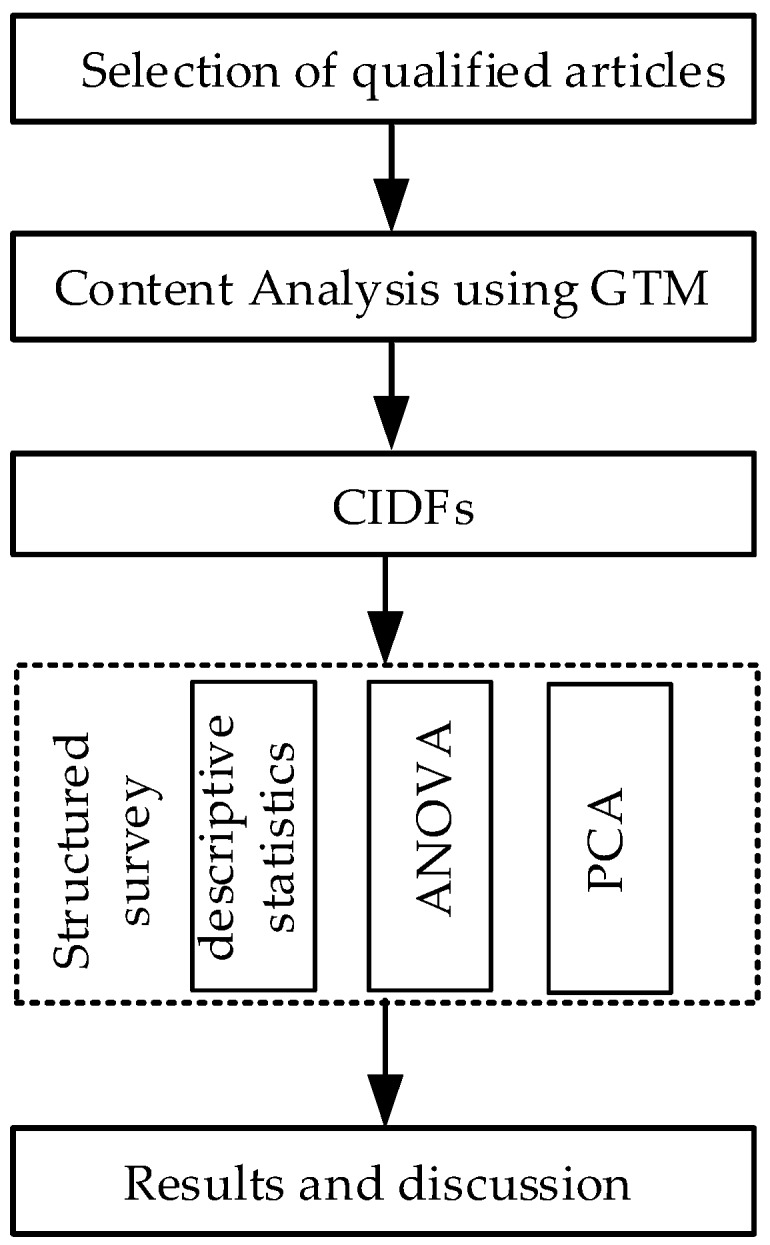
The research roadmap. GTM: grounded theory method, CIDFs: construction industrialization driving factors, PCA: principal component analysis.

To explore the driving factors of CI development in China, this paper combined both qualitative and quantitative research. The grounded theory method (GTM) was employed to conduct a content analysis based on qualified CI-related articles from selected journals. A structured questionnaire survey, investigating respondents’ attitudes towards the identified CIDFs, was conducted in Jiangsu province, which can represent the development of construction industry in China, especially the development of CI at the initial stage. Descriptive statistics, analysis of variance (ANOVA), and principal component analysis (PCA) helped the questionnaire analysis. Finally, the results of both the qualitative research and the quantitative research were discussed. The roadmap of this research is shown in Figure 1.

A systematic review of publications would help researchers to gain in-depth insights into the advancement in a selected field [25]. By analyzing the content of previous research, the motivations and incentives could be identified [26]. In this paper, the content analysis was conducted in order to systematically and objectively explore CIDFs on the basis of large volumes of published materials [26,27]. To obtain qualified publications for content analysis, the literature selection method employed by Li et al. [12], and Ke et al. [28] was adopted. This research first conducted a general search with the help of Scopus search engine in the field of construction management. The search strategy was to explore articles containing the term “CI” and the above-mentioned synonymous terms in the title/abstract/keywords, from 2000 to the end of 2017. The results of the first-round search showed that a large number of CI-related articles were published in various journals. To ensure the quality and improve the efficiency of this research, a second-round search was narrowed to journals with more than 5 CI-related articles that were ranked by Wing [29] as first-tier journals. Finally, a total of 182 CI-related journal articles were obtained for further analysis. The list of journals and the distribution of these academic articles are shown in Table 1.

The grounded theory was initially developed by Glaser and Strauss in the 1960s to help generate a theory based on empirical research [30]. Rooted in medical sociology, the grounded theory method (GTM) has been applied in various research fields, such as nursing, education, psychology, sociology, etc. It is efficient to study complex topics with limited information [31]. In GTM, data analysis follows a well-defined process that begins with a basic description and moves to conceptual ordering and then to theorizing [32]. Coding is the core process of GTM, which can be divided into three basic phases, i.e., open, axial, and selective coding [33]. In this paper, GTM was employed as an efficient content analysis tool to make an exploratory research to study the incentives of CI development in the selected articles. As a result, a number of CIDFs would be obtained for further study.

Questionnaire survey is an effective method to obtain the respondents’ view about a selected topic [6]. The data collected were analyzed with the help of statistical techniques. The Statistical Package for the Social Science (SPSS, IBM Corporation, Armonk, NY, USA) was employed in this paper to test the reliability of the collected data and conduct descriptive statistics, i.e., ANOVA and principal component analysis (PCA).

## 3. CIDFs Exploration Based on the Ground Theory Method (GTM) 

### 3.1. Emergence of Concepts

The data was gathered through a literature review and was coded using the open coding method. Open coding is the interpretive process by which data is broken down analytically into abstract concepts. The separate concepts were examined and compared carefully for similarities and differences. Key points from the literature were grouped, and 14 concepts emerged (Table 2).

### 3.2. Emergence of Categories

It can be seen that some of the concepts share common or similar characteristics and can be grouped at a higher level. Thus, the concepts were grouped into three core categories: external environment, transformation and upgrade of the construction industry, and strategy of the government, as shown in Table 3. The concepts and categories that emerged from the literature review formed the basis for the subsequent structured questionnaire survey.

### 3.3. Factor Group Description

According to the findings of GTM-based exploration, 14 CIDFs were identified as follows:

- F1 Demands for more production
- F2 Lack of labor
- F3 Serious environment problems
- F4 Shortage of resources
- F5 Technologies progress
- F6 Integration of advanced technologies
- F7 Management improvement
- F8 Productivity improvement
- F9 Quality improvement
- F10 Cost reduction
- F11 Construction-time reduction
- F12 Integration of the supply chain
- F13 Health and safety improvement
- F14 Supporting policies

These identified factors represent the driving forces which put forward the development of CI. The 14 driving forces mainly come from the following three categories.

The first category of the CIDFs reflects the exogenous driving forces represented by the dilemmas and progress of the external environment development. This category includes F1, F2, F3, F4, F5, and F6. These factors outline current macroenvironment for the development of CI. First, the rapid urbanization in China has brought masses of people to the urban areas, thus increasing the demands for large amounts of dwellings for the citizens (F1). The adoption of CI in China is expected to fill the gap between rapid population growth and shortage of production. During the urbanization movement, the craft-based conventional construction industry is also facing a serious shortage of workforce, especially of skilled craftsmen (F2). The harsh construction conditions are no longer attractive to the new generation of migrant workers. In the meantime, the development of the society is facing serious constraints from the fragile environment (F3) and limited resources (F4). Methods employed in the traditional construction industry have consumed great amounts of resources and generated huge quantities of waste, which have caused severe damages to the environment. Recently, technologies including BIM (building information modeling) and RFID (Radio-frequency identification) have been developing at a remarkable speed (F5). The application and integration of these advanced technologies (F6) help to transform the conventional construction industry into industrialized construction. To sum up, it is expected that the adoption of CI would take advantage of these progresses and relieve the dilemmas of the macrodevelopment. Thus, the first category shows that the adoption of CI is pushed by the external environment.

The second category of the CIDFs represents the endogenous driving forces of the demands for transformation and upgrade within the construction industry. The traditional construction industry has been blamed for a low level of management (F7) and productivity (F8), unstable quality (F9), budget overrun (F10), schedule delay (F11), separate supply chain (F12), and health and safety threats (F13) for years. To change the status quo, it is urgent to introduce a new philosophy and new methods into the construction industry. CI transfers parts of the construction activities and components into the factory, where the working conditions are more controllable, and then transports components to the construction site for their final assembly. The adoption of CI makes it possible to transform methods and principles used in the manufactory industry so to transfer them into the construction industry. For example, the introduction of the lean principle and just-in-time delivery has helped to integrate the supply chain of the construction industry [41]. By taking advantage of these methods and principles, the construction industry could realize the its own transformation and upgrade. This category indicates that the adoption of CI is a self-driven approach.

**Figure 2:**
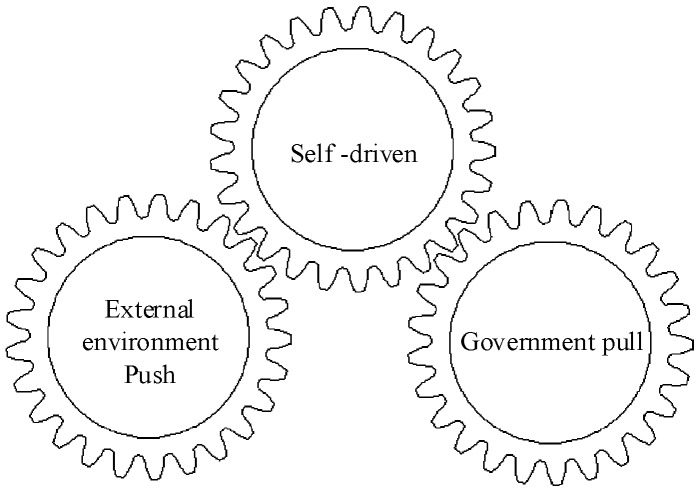
Driving forces of CI development in China.

The third category of the CIDFs also represents external driving forces represented by the support from the government. In most cases, the government is responsible for the sustainable development of society, and the construction industry is not an exception. With its the top-level design to solve the development dilemmas and to transform and upgrade the construction industry, CI is supported by the government. Both the central and local government have established supporting policies to guide and supervise the development of CI in China (F14), and resemble an invisible pulling force to accelerate the realization of CI [42]. The three driving forces of CI development are illustrated in Figure 2.

## 4. Research Survey

### 4.1. General Information on the Survey

A structured questionnaire survey was conducted from September to December 2017 to study the identified driving factors and obtain views of the respondents on the development of CI in Jiangsu, China, which is the forerunner among all the regions in the development of both macroeconomy and construction industry. For example, the total output value of Jiangsu construction industry in 2016 was approximately 2954.9 billion CNY (434.5 billion USD), which corresponded to 13.3% of the total output value of the national construction industry [43]. Numerous indices of Jiangsu construction industry have been the highest among all the provinces for years, including the total output value, number of construction companies, employees etc. [43]. Therefore, the development of the construction industry in Jiangsu could be considered as a microcosm for the development of the construction industry in China. Besides, this region was selected as the first pilot province by the central government to promote CI. The questionnaires were sent to people from the government, enterprises, research institutions such as universities, and to citizens in Jiangsu province.

Before the formal survey, the questionnaire was pre-tested to ensure that the questions were clear, practical, and not overly burdensome to answer. The initial draft was sent to CI scholars and senior officers in the Department of Housing and Urban–Rural Development (DoHURD) of Jiangsu province who are responsible to make policies for CI development in Jiangsu province. According to their feedback on the test questionnaire survey, the respondents suggested that pilot programs of CI could play significant roles in promoting CI and should be taken into consideration. Therefore, *Pilot programs* was added as the fifteenth CIDF.

The final questionnaire comprised two sections. The first section was about the respondents’ background information such as age, education, occupation, and working experience. The second section investigated the respondents’ views on the 15 driving factors using a five-point Likert scale. The scale intervals are interpreted as follows: (1) Can be ignored or not important; (2) Possibly important; (3) Important; (4) Quite important; (5) Most important. A total of 200 questionnaires were sent, and 123 were reclaimed, with 61.5% valid response rate. The distribution and general information of the respondents are listed in Table 4.

Statistics showed a wide range of respondents from different stakeholders related to the construction industry, including 24 government officers, 57 staffs from the construction enterprises, 25 researchers, and 17 citizens. Their ages were mainly between 21 and 40 years; people between 41 to 50 years old represented a significant proportion of respondents. The distribution of the respondents’ occupation and age showed a decent coverage of these variables in the respondents. In addition, over 95% of the respondents held a bachelor's degree or above, and a large proportion had a working experience of more than 5 years in the construction field. The educational background and working experience of the respondents indicated that they had a good awareness of CI development, which further ensured the reliability of the survey.

### 4.2. Ranking of the Driving Factors

A descriptive statistical analysis can provide basic but fundamental information about the results reflecting the opinions of the respondents on the driving factors. Cronbach’s Alphas was used to test the reliability of the questionnaire survey, and the result was 0.827, which indicates a high consistency of the collected data [44]. The result of the descriptive analysis is listed in Table 5. The mean values of the 15 driving factors ranged from 4.26 (F15, Pilot programs) to 3.93 (F13, Health and safety). Most of them were above 4, which revealed that the identified factors were considered to be of high importance by the respondents to put forward CI development.

As shown in Table 5, pilot programs (F15) occupies the first place among all these driving factors, which reflects that pilot projects and demonstrations of CI had a strong influence on the respondents’ perceptiveness. In fact, all the stakeholders expressed considerable concerns about the performance of CI pilot programs during the promotion period. F14 in the third category, which also reflects the attitudes of the government, also ranks in the front.

The ranks of F2, F4, and F3 are relatively higher in the first category. These three factors reflect the bottleneck for sustainable development in China, especially within the traditional construction industry. While the craftsmen who supported the rapid growth of the traditional construction are aging, the construction industry is no longer attractive for the new generation of migrant workers. What is worse, China is becoming an aging society, which pushes the construction industry to adopt the production method of the manufacturing industry. Besides, the limited resources and fragile environment also hinder the development of the conventional construction industry. Despite the fact that the demands for a higher production (F1) were not considered so important in this survey, compared with the trajectories of CI development in other regions [4,8,13], these demands during the process of urbanization provide great opportunities for the development of CI in China. A low level of technology application has been blamed for years [45]. Therefore, F6 and F5 rank at the bottom in the first category. In fact, the technologies currently applied in the construction industry have been invented or deployed in the manufacture industry for years. Therefore, technology progress was not considered of high importance.

In the second category, the ranks of F8, F9, and F7 are higher than those of the other three factors. The demand to increase the productivity (F8) was listed as the top priority of transformation and upgrade within the construction industry. It reflects the original intentions of CI, which is to adopt the model of the manufacturing industry and realize mass production. Quality improvement (F9) was also given high importance by the respondents. In the conventional construction industry, the quality of products is unstable and affected by numerous factors, such as the weather, workers’ proficiency etc. Through the adoption of CI, the work environment would become more controllable, advanced technologies could be employed to replace the construction workers in some process, and new philosophies of management could also be introduced to improve product quality. Besides, new management methods would also improve the management level of the construction industry (F7) and help to integrate the supply chain (F12), factors that also received much concern from the respondents. The reduction of construction schedules (F11) and costs (F10) were considered less important in promoting CI. In addition, the concern for health and safety (F13) was regarded as the least important driving factor in China.

Overall, the descriptive analysis showed general information about the attitudes toward CI driving factors. Currently, the driving forces from the Chinese government are leading the development of CI. In the external environment, dilemmas regarding the development rather than the technological processes are more significant in pushing the development of CI. Within the construction industry, the transformation and upgrade for higher productivity, higher quality, and management improvement are the main endogenous factors driving CI development.

### 4.3. Agreement on the Driving Factors

The analysis of variance (ANOVA) was conducted to explore the agreement on the driving factors of the different respondents (government officers, staffs from the enterprises, researchers, citizens). The result of ANOVA is presented in Table 6. While the significance of most factors was above 0.05, only for three factors, i.e., F10 (0.045), F13 (0.018), and F14 (0.010) it was lower than 0.05. According to Bea, et al. [46], the respondents of different groups reached agreement on most factors but held different views on the reduction of construction cost (F10), concerns for health and safety (F13), and supporting policies offered by the government (F14).

Currently, the adoption of CI at this stage has been limited to the pilot programs, and the reduction of construction cost by mass production has not been realized. In previous research, the construction cost would rise at the initial stage of CI development [1,3,12]. In fact, the promotion of CI in the pilot programs is supported by subsidies from the government. The different groups also disagreed about F14. While the adoption of CI transfers some construction activities into the factory, there still remains a lot of work to be completed on the construction site. For example, after being prefabricated in the factory, the components need to be transported to the construction site by trucks, have to be lifted by cranes, and finally have to be assembled at a certain height. More evidence of the alleviation of health and safety risks with the development of CI should be accumulated to reach agreements by all parties. Compared to the pilot programs (F15), these supporting policies mostly appear in the documents offered by government or professional institutions. Therefore, the respondents without professional knowledge might have failed to understand the incentives.

Factors reflecting the dilemmas of the macrodevelopment (F2, F3, and F4), the development of CI, and the demands for more production (F1) were approved by all groups, and meanwhile, they also acknowledge the contributions of technologies (F5) and their integration (F6) in the first category. In the second category, different groups reached higher agreement on the improvement of productivity (F8) and management (F7) and on the reduction of construction time (F11). The improvement of quality (F9) and the integration of supply chain (F12) were also accepted by major respondents of different groups as important in driving the development of CI in China.

### 4.4. Exploratory Factor Analysis 

As depicted above, the obtained CIDFs could be grouped into three categories, and it was necessary to conduct an exploratory factor analysis to verify the hypothesis in the context of China. Exploratory factor analysis helps to explain these variables according to their common underlying dimensions by condensing the information contained in a number of original variables into a smaller set of dimensions with a minimum loss of information [47]. In addition, factor analysis can also calculate the relative significance of these dimensions. The collected data of the respondents were subjected to this technique to determine whether or not groupings of the driving factors of CI development in China could be established.

Before the factor analysis, the Kaiser–Meyer–Olkin (KMO) test and Bartlett’s Sphericity test were employed to examine the adequacy of the factor analysis for the collected survey data. The results of KMO and Bartlett’s Sphericity tests are shown in Table 7. The value of KMO test was 0.795, which indicated a high level of data adequacy for factor analysis [48]. The value of the significance test was 0.000, i.e., lower than 0.001, which indicated strong correlations between the variances. The results of the validity test showed that the acquired data was valid and could be used for factors analysis.

The principal components and varimax rotation were used to uncover the interrelationships among the 15 driving factors. The factors that were highly correlated were separated into a small number of major components (dimensions). Three principal components whose eigenvalues were greater than 1 were extracted in the exploratory factor analysis. As shown in Table 8, three obtained components cumulatively explained 68.71% of the total variances. Although the cumulative percentage was a bit lower, according to Jolliffe [47] principal component analysis (PCA), as an exploratory factor analysis, the model with a three-factor grouping was acceptable to represent the data.

The results of the rotated component matrix are shown in Table 9. To make it easier to read, absolute values less than 0.5 were suppressed. Each row of Table 9 contains component loadings, which indicate the correlations between each variable and component (the dimension of the driving factors). The first component has the largest variance and therefore can explain the problem most effectively. The second component is independent from the first component and contains as much of the remaining information, which reflects the driving forces from the macroenvironment. The third component reflects the pulling force from the government. The result of the exploratory factor analysis is consistent with the grouping of GTM.

F7, F10, F8, F9, F11, F13, and F12 were clustered into the same group, and are the self-driven factors. Factors of F5, F6, F3, F4, F1, and F2 were grouped into the second group, named “Macroenvironment push”. F15 and F14 were aggregated in the third group, named “Government pull”. The results of the factor analysis verified the findings of the previous section obtained by GTM based on the literature review.

## 5. Discussion

The results of the GTM-based exploration identified 14 CIDFs based on published international journal articles. Pilot programs, which play significant roles in promoting CI development in China, were added as the 15th CIDF in this paper. The identified factors were grouped into three categories, namely, external development, transformation and upgrade of the construction industry, and strategies selected by the government. These three categories represent three major driving forces that put forward the development of CI in China. That is, the development of CI is not only pushed by the macro-development or pulled by the government, but it is also a self-driven process.

The identified CIDFs in the qualitative exploration were further studied by a structured questionnaire survey. The results of PCA confirmed the grouping by the GTM-based exploration. According to PCA, the dimension of the self-driven factors has the largest variance, which means that the respondents considered the transformation and upgrade within the construction industry as the most fundamental demand to promote CI in China, followed by the driving forces from the external development. The promotion of CI in China also relies on the supporting strategies of the government.

Among the 15 CIDFs, pilot programs set up by the government were considered to be the most important in CI promotion. Pilot programs directly demonstrate the merits of CI to the public, which would help to increase awareness and acceptance by the society. In addition, pilot programs would also accumulate valuable experience for the future development of CI. More importantly, pilot programs reflect the willingness of the Chinese government to promote CI development, which is of great importance to motivate the participation of other stakeholders, especially at the initial stage. Compared to the advancement and integration of technologies, development dilemmas, including labor shortage, environment problems, and resource shortage play more significant roles in pushing CI development in China. In fact, most technologies recently adopted in construction industry have been developed for years. These dilemmas have hindered the sustainable development of the society and also the construction industry. Therefore, the adoption of CI is expected to meet these constraints while providing a higher construction production for the society. Within the construction industry, the improvement of productivity, quality, and management are considered as the priority incentives to promote CI in China. Currently, with the rapid urbanization of China, a higher quality production is needed in major cities. To achieve such goals, the traditional extensive methods of management are no longer able to meet the current requirements. A new management philosophy, such as lean construction, should be introduced to improve the management in the construction industry. Integration of the supply chain is also a major concern within the construction industry. Currently, different phases and stakeholders are separated, which leads to inefficiency and wastage in the entire industry. Through the implementation of CI, the supply chain would be more integrated, and the lifecycle performance of projects would be improved. However, the adoption of CI in China is still at the initial stage, construction workers are not familiar with the new method, and mass production has not been realized. Therefore, CI has not fully demonstrated its advantages, such as time and cost reduction. In fact, according to the data collected in the pilot projects, the cost is 10% higher for CI projects than for the conventional projects. The promotion of CI is still relying on subsidies from the government. In the meantime, since CI has not been widely adopted, there is no direct evidence of the link between CI adoption and the decrease of health and safety risks. Therefore, the improvement of health and safety has not been acknowledged by the respondents as a major motivation in promoting CI in China.

The results of ANOVA showed that respondents with different background reached agreements on most CIDFs. The development of CI in China is the resultant of external environment push, self-driven, and government pull process. However, the respondents held different views on cost reduction, health and safety improvement, and policies offered by the government. As depicted above, CI promotion is still at the initial stage, and people without the expertise in this field have not perceived CI merits yet. It is necessary to collect data in pilot projects and advocate these benefits to the public, which would improve their understanding and thereby help the promotion of CI in China. Besides, the supporting policies offered by the government usually appear in the form of government documents, which are rigid for the ordinary citizens to understand comprehensively. The government should make these policies more comprehensible to reach the public. The divergence also reflects the concerns about the effects of these promotion policies. The government should strengthen the top-level design to promote CI adoption and make feasible plans for the successful applications of CI in China.

## 6. Conclusions

The development of CI has gain extensive interest in China because of its potential benefits. While different stakeholders are pursuing their own goals, it is critical to conduct a systematical analysis of the driving factors for the decision makers to understand the incentives of CI development and help them to select proper strategies. This study combined both qualitative and quantitative methods to identify the CIDFs in China. GTM was employed to explore the driving factors of CI development from 182 selected CI-related articles in the field of construction management. These articles were published from 2000 to 2017 in ten top-tier academic journals, including AIC, CME, JCEM, EB, JAE, CI, BE, ECAM, HI, and BRI. A total of 14 general CIDFs, which put forward CI adoption in different regions of the world, were identified. A structured questionnaire survey was conducted to investigate the respondents’ views about the identified CIDFs. One specific driving factor was supplemented by professionals during the pre-test questionnaire survey. Finally, 15 CIDFs were included in the questionnaire survey.

The GTM-based qualitative exploration showed that the driving forces of CI development mainly come from three categories, namely, external environment push, self-driven processes, and government pull. The results of the survey showed that all the 15 identified CIDFs are of importance for the adoption of CI in China. Specifically, the transformation and upgrade of the construction industry is the fundamental incentive for the development of CI. Besides, during the promotion of CI, the decision makers should take the development dilemmas, such as labor shortage, environment degradation, and resource constraints, into consideration. To promote CI, pilot programs are the most effective way in China. This suggests that decision makers could take full advantage of pilot programs as platforms to gather field data and demonstrate CI to the public.

This study is also subjective to limitations. One of them is that to ensure the quality of the articles, only 10 journals were selected. In addition, the development of CI in China is still at its initial stage. Some of the benefits have not been realized, for example, cost reduction and improvement in health and safety performance. More evidence should be accumulated to gain recognition of CI importance by the society.

## Figures and Tables

**Table 1:** Journal list and the distribution of Construction industrialization (CI)-related articles.

	Name of Journals	Abbreviation	No. of Articles
1	Automation in Construction	AIC	40
2	Construction Management and Economics	CME	33
3	Journal of Construction Engineering and Management	JCEM	25
4	Energy and Building	EB	20
5	Journal of Architectural Engineering	JAE	19
6	Construction Innovation	CI	12
7	Building and Environment	BE	11
8	Engineering, Construction, and Architectural Management	ECAM	9
9	Habitat International	HI	8
10	Building Research and Information	BRI	5

**Table 2:** Results of open coding.

Concepts	Descriptions of the Data
Demands for more production	The purpose of this paper is to establish manufactured construction as a good potential alternative to meet the growing housing needs of China [34].
Lack of labor	Prefabrication has been increasingly adopted in the delivery of housing plans to alleviate various constraints, such as labor shortage [35].
Serious environment problems	There is an urgent issue regarding the huge quantities of wastage generation during construction. There should not be lack of environmental support from construction stakeholders [15].
Shortage of resources	It is shown that there is significant potential for the reuse of materials in the prefabricated steel building, representing up to an 81% saving in embodied energy and 51% materials saving by mass [36].
Technologies progress	Technologies have played a major role in the process of forming the architectural theory and practice during the twenty-first century [37].
Integration of advanced technologies	Internet of Things (IoT)-enabled platform deploying building information model (BIM) to re-engineer offshore prefabricated construction processes [35].
Management improvement	Emergent organizational change strategies like rewarding innovation and fostering bottom-up communication are still underdeveloped [21].
Increase productivity	An especially strong and significant positive correlation is found to exist in resource development, worker involvement, process improvement, and task recognition as they refer to off-site construction productivity [38].
Quality improvement	Quality audits from three phases of the building process were compiled, analyzed, and categorized to provide statistical measures of defects in industrialized housing. The results show that the case study companies were better in terms of product quality than conventional housing [39].
Cost reduction	The spending of off-site construction (OSC) was lower, and the shift from on-site construction to factory-based indoor prefabrication decreased the number of workers required and the project delivery timeframe, thereby contributing to cost savings [24].
Time reduction	OSC may offer the opportunity to help fulfill the need by significantly reducing the construction time [22].
Integration of supply chain	Earlier engagement with supply chains was advocated for favoring the off-site approach and improving business efficiency [23].
Health and safety improvement	This paper presents the results of an interview survey of major construction clients about their expectations from drivers for pre-assembly on their projects. Improved health and safety along with other merits were identified [4].
Supporting policies	It is the only major client in Hong Kong requiring prefabrication in its public housing construction, a policy which began in the mid-1980s [40].

**Table 3:** Result of axial coding.

Core Categories	Concepts	Meanings
External environment	Demands for more production	Pressures for more dwellings through the adoption of CI
	Lack of labor	Labor shortage in the working force market, especially of skilled construction workers
	Serious environment problems	Environmental damage and wastage generated in the construction industry
	Shortage of resources	Resources constraint the development of the conventional construction industry
	Technologies progress	The development of technologies, including information technologies and advanced construction techniques in construction industry
	Integration of advanced technologies	The integration of advanced technologies helps the development of CI
Transformation and upgrade of the construction industry	Management improvement	Employ new management philosophies in construction industry
	Increase productivity	Boost the productivity through the manufactured process
	Quality improvement	Improve the quality of construction products through the factory
	Cost reduction	Reduce the life-cycle cost of the project through standardized design, components, etc.
	Time reduction	Increase the speed and reduce schedule delays
	Integration of the supply chain	Integrate different phases and various stakeholders in the life cycle of construction projects
	Health and safety improvement	Improve the health and safety performance through the adoption of CI
Strategy of the government	Supporting policies	Support policies established by the government

**Table 4:** Information about the respondents.

Role	Government Officers	Staffs from Enterprises	Researchers	Citizens	N/A	Total
Number	24	57	25	17	-	123
percentage	19.5%	46.3%	20.3%	13.8%	-	100%
Age	21 to 30	31 to 40	41 to 50	51 to 60	Over 60	Total
Number	47	51	21	4	0	123
percentage	38.2%	41.5%	17.1%	3.3%	0%	100%
Educational background	college	undergraduate	postgraduate	N/A	N/A	Total
Number	6	73	44	-	-	123
percentage	4.9%	59.3%	35.8%	-	-	100%
Working experience	5 years or under	6–10 years	11–15 years	16–20 years	Over 20 years	Total
Number	22	60	28	10	3	123
percentage	17.9%	48.8%	22.8%	8.1%	2.4%	100%

**Table 5:** Results of the descriptive statistical analysis.

Factors	Min	Max	Mean	SD	Rank
F15	2	5	4.26	0.734	1
F2	1	5	4.20	0.846	2
F4	2	5	4.20	0.765	2
F8	1	5	4.20	0.765	2
F3	2	5	4.16	0.670	5
F9	2	5	4.16	0.740	5
F14	2	5	4.16	0.717	5
F7	1	5	4.11	0.812	8
F12	2	5	4.11	0.755	8
F1	2	5	4.06	0.761	10
F11	2	5	4.02	0.849	10
F6	1	5	4.01	0.835	12
F10	2	5	3.99	0.719	13
F5	2	5	3.98	0.849	14
F13	2	5	3.93	0.817	15

SD: standard deviation.

**Table 6:** Results of ANOVA.

Groups Description		Sum of Squares	df	Mean Square	F	Significance
F1	Between Groups	3.082	3	1.027	1.811	0.149
	Within Groups	67.520	119	0.567		
	Total	70.602	122			
F2	Between Groups	2.043	3	0.681	0.950	0.419
	Within Groups	85.274	119	0.717		
	Total	87.317	122			
F3	Between Groups	0.472	3	0.157	0.345	0.793
	Within Groups	54.276	119	0.456		
	Total	54.748	122			
F4	Between Groups	0.866	3	0.289	0.488	0.691
	Within Groups	70.451	119	0.592		
	Total	71.317	122			
F5	Between Groups	1.461	3	0.487	0.670	0.572
	Within Groups	86.506	119	0.727		
	Total	87.967	122			
F6	Between Groups	1.729	3	0.576	0.824	0.483
	Within Groups	83.263	119	0.700		
	Total	84.992	122			
F7	Between Groups	2.121	3	0.707	1.075	0.363
	Within Groups	78.285	119	0.658		
	Total	80.407	122			
F8	Between Groups	1.151	3	0.384	0.667	0.574
	Within Groups	68.475	119	0.575		
	Total	69.626	122			
F9	Between Groups	2.689	3	0.896	1.665	0.178
	Within Groups	64.059	119	0.538		
	Total	66.748	122			
F10	Between Groups	5.741	3	1.914	2.770	0.045
	Within Groups	82.226	119	0.691		
	Total	87.967	122			
F11	Between Groups	1.692	3	0.564	1.095	0.354
	Within Groups	61.300	119	0.515		
	Total	62.992	122			
F12	Between Groups	5.220	3	1.540	2.132	0.128
	Within Groups	66.097	119	0.555		
	Total	71.317	122			
F13	Between Groups	6.587	3	2.196	3.489	0.018
	Within Groups	74.893	119	0.629		
	Total	81.480	122			
F14	Between Groups	5.722	3	1.907	3.980	0.010
	Within Groups	57.026	119	0.479		
	Total	62.748	122			
F15	Between Groups	2.394	3	0.798	1.501	0.218
	Within Groups	63.280	119	0.532		
	Total	65.675	122			

**Table 7:** Results of the Kaiser–Meyer–Olkin (KMO) test and Bartlett’s Sphericity test.

Index	KMO Measure of Sampling Adequacy	Bartlett’s Sphericity Test		
		Chi-Square Value	Degree of Freedom	Significance
value	0.795	469.567	105	0.000

**Table 8:** Total Variance Explained by the Principal Component Analysis.

Component	Initial Eigenvalues			Rotation Sums of Squared Loadings		
	Total	% of Variance	Cumulative (%)	Total	% of Variance	Cumulative (%)
1	4.475	29.83	29.83	4.053	27.02	27.02
2	3.480	23.20	53.03	3.295	21.97	48.99
3	2.352	15.68	68.71	2.959	19.72	68.71

**Table 9:** Rotated Component Matrix for the Total 15 Driving Factors.

Factor Groupings	Driving Factors	Components		
		1	2	3
Self-driven	F7	0.912	-	-
	F10	0.815	-	-
	F8	0.730	-	-
	F9	0.715	-	-
	F11	0.658	-	-
	F13	0.625	-	-
	F12	0.534	-	-
Macro-environment push	F5	-	0.863	-
	F6	-	0.756	-
	F3	-	0.642	-
	F4	-	0.574	-
	F1	-	0.550	-
	F2	-	0.527	-
Government pull	F15	-	-	0.779
	F14	-	-	0.508
